# Mendelian Randomization Reveals Interleukin-6 Receptor-Lipoprotein(a) Interplay With Independent Cardiovascular Risk Reductions

**DOI:** 10.1016/j.jacbts.2026.101564

**Published:** 2026-05-08

**Authors:** Azin Kheirkhah, Silvia Di Maio, Stefan Coassin, Sebastian Schönherr, Claudia Lamina, Lukas Forer, Florian Kronenberg, Johanna F. Schachtl-Riess

**Affiliations:** Institute of Genetic Epidemiology, Medical University of Innsbruck, Innsbruck, Austria

**Keywords:** cardiovascular disease, interleukin-6 receptor, lipoprotein(a), Mendelian randomization

## Abstract

In the study at hand, 2-sample Mendelian randomization supported a causal effect of interleukin-6 receptor (IL-6R) signaling on Lp(a) concentrations, revealing a direct interplay between 2 causal risk factors for cardiovascular disease (CVD). Importantly, epidemiological analyses in UK Biobank showed that neglecting the IL-6R functional state can confound the association between IL-6 levels and Lp(a). However, the effect of circulating IL-6 on Lp(a) concentrations was modest even when the functional state of the receptor was considered. In line, factorial Mendelian randomization indicated that down-regulation of IL-6R signaling and Lp(a) independently reduce CVD risk, highlighting complementary opportunities for CVD risk mitigation.

## Highlights

• IL-6R signaling and Lp(a) are causal CVD risk factors; IL-6R inhibitors lower Lp(a) but raise circulating IL-6 concentrations.
• Two-sample MR supports a causal effect of IL-6R signaling on raising Lp(a).
• Ignoring IL-6R functional state can confound IL-6–Lp(a) associations in epidemiological analyses.
• Factorial MR shows genetically lowered IL-6R signaling and Lp(a) independently reduce CVD risk.

## Visual Abstract

**Figure:**
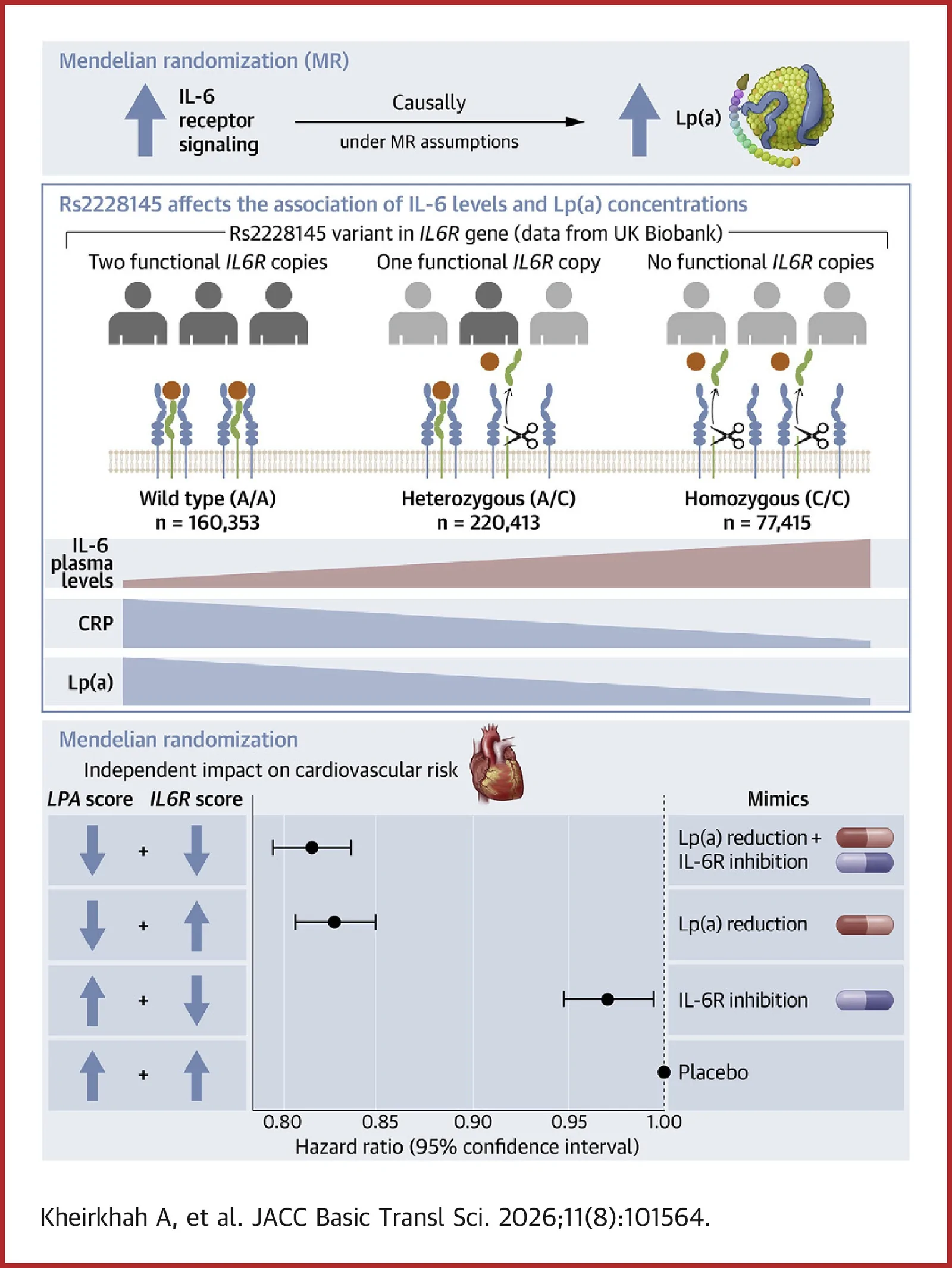

Cardiovascular disease (CVD) is a leading cause of death worldwide.^1^^,^^2^ New approaches in prevention and treatment are essential for decreasing its global burden.^3^^,^^4^ Atherosclerosis, a major underlying CVD cause, is a complex disease largely driven by an interplay between lipids and inflammation.^5^, ^6^, ^7^ In the past decade, nontraditional risk factors including lipoprotein(a) [Lp(a)] and inflammatory cytokines such as interleukin (IL)-6 have gained attention as emerging therapeutic targets.^8^, ^9^, ^10^, ^11^

IL-6 is a downstream mediator of the IL-1B signaling pathway. IL-6 binds to either a membrane-bound interleukin-6 receptor (IL-6R) or a soluble IL-6R,^12^ increasing transcription of downstream molecules like C-reactive protein (CRP). Several lines of evidence showed an association between IL-6 levels and CVD risk.^13^, ^14^, ^15^ Mendelian randomization (MR) studies supported causality of IL-6R signaling in CVD risk by showing that the loss-of-function (LOF) variant rs2228145 (Asp358Ala) in the gene encoding for the IL-6R (*IL6R*) reduced CVD risk.^16^^,^^17^ Both IL-6 and IL-6R inhibitors down-regulate IL-6R signaling. Treatments with IL-6 inhibitors are currently investigated in clinical trials for CVD.^8^^,^^9^

Similarly, clinical trials targeting elevated Lp(a) have reached phase 3.^10^^,^^11^ Lp(a) is the lipoprotein with the strongest genetic control,^18^ and MR studies supported its causality in CVD.^19^, ^20^, ^21^ Although the variance in Lp(a) concentrations is almost entirely determined by the *LPA* gene, some fluctuations stem from hormones,^18^ liver, and kidney disorders,^19^^,^^22^ but also infections and chronic inflammatory diseases.^23^^,^^24^

The *LPA* promoter region includes response elements for IL-6,^25^ and several studies suggest an impact of the IL-6 axis on the Lp(a) concentrations.^26^^,^^27^ Recently, genetic variants in the *IL6* locus mimicking IL-6 inhibition showed Lp(a)-lowering effects.^28^ In clinical trials of high-risk populations, medications blocking either IL-6 or -6R reduced Lp(a) levels by 16% to 41%.^29^ However, down-regulation of IL-6R has been associated with higher circulating IL-6 levels.^16^^,^^30^ This urges the decoupling of circulating IL-6 from the IL-6R functional state in understanding downstream signaling and its effect on Lp(a) concentrations.

Moreover, there is ongoing debate that the association between Lp(a) and CVD risk is only present when there is a residual inflammatory risk, marked by elevations of IL-6^31^, ^32^, ^33^ and CRP.^34^, ^35^, ^36^, ^37^, ^38^, ^39^, ^40^ However, the extent to which IL-6R signaling modulates Lp(a) concentrations at population level remains undefined, leaving the implications of their potential interplay for CVD treatment unclear.

In this study, we sought to test the potential causal role of IL-6R signaling in Lp(a) concentrations using MR. Moreover, we use the UK Biobank (UKB) to investigate the relevance of this mechanistic link at the population level, decoupling IL-6 levels from IL-6R activity. Finally, we investigate whether genetic variants related to IL-6R signaling and Lp(a) are independently associated with CVD.

## Methods

### Study description

We utilized several publicly available summary data, with study metadata and access details provided in the Supplemental Appendix. For UKB, we additionally analyzed individual-level data, which are described here in greater detail. UKB is a prospective cohort study with approximately 500,000 participants recruited between 2006 and 2010 (age at enrollment 40-69 years). The study was approved by the North West Multi-centre Research Ethics Committee. Genotyped and imputed data were generated as described elsewhere.^41^ Lp(a) concentrations and CRP levels were measured at recruitment with immunoturbidimetric assays, while IL-6 plasma levels (rank inverse normally transformed protein levels) were provided as part of the proteomic profiling conducted in 54,219 UKB participants using the antibody-based Olink Explore 3072 proximity extension assay.^42^ Here, we restricted the analyses to participants of self-reported White ancestry. In total, 48,780 participants experienced CVD events (Supplemental Table 1). Further details on UKB genetic and phenotypic data are in the Supplemental Methods.

### Instrumental variables

Gene-locus–specific instrumental variables at the *IL6R* and *LPA* loci were used for MR for the following: 1) to investigate if IL-6R signaling has a causal effect on Lp(a) concentrations (Lp(a) concentrations = outcome); and 2) investigate if down-regulation of IL-6R signaling and Lp(a) concentrations have an independent effect on CVD risk (CVD = outcome). Detailed descriptions of the instrumental variables are provided in the Supplemental Methods.

We used single-nucleotide variants (SNVs, formerly SNPs) in the *IL6R* locus as instrumental variables for IL-6R signaling (=exposure, proxied by CRP concentrations, a known downstream biomarker of IL-6R signaling) (Supplemental Table 2). They have been validated to mimic the effect of IL-6R signaling and its inhibition (eg, change in CRP, circulating IL-6, and soluble IL-6R levels) and have been described before in detail.^43^^,^^44^ In epidemiological analyses we used the known LOF variant rs2228145 to proxy IL-6R functional state. The minor allele (rs2228145-C) increases proteolytic cleavage of the membrane-bound IL-6R, thereby down-regulating IL-6R signaling.^45^^,^^46^ The minor allele frequency in 1000 Genome populations is provided in Supplemental Table 3.

As instrumental variables for Lp(a) concentrations (=exposure), we used a previously described weighted score of 43 SNVs in the *LPA* region^47^^,^^48^ (factorial MR) as well as 14 SNVs from a meta-GWAS in 13,781 individuals (reverse 2-sample MR)^49^ (Supplemental Table 4).

As indicated in the respective Statistical Analysis and Results sections, SNVs were either taken as separate instrumental variables or combined into scores and calculated for UKB using pgs-calc (version 1.6.1).^50^

Of note, all MR analyses rely on the instrumental variable assumptions: 1) relevance, the instruments are associated with the exposure (assessed via F-statistics for single SNVs [Supplemental Tables 2 and 4] and variance explained for scores [Supplemental Methods]); 2) independence, instruments are independent of unmeasured confounders of the exposure–outcome relationship; and 3) exclusion restriction, instruments affect the outcome only through the exposure (assessed with robust methods).^51^

### Statistical analysis

Statistical analyses were performed in UKB White participants (n = 471,607) and in a subset with available IL-6 and Lp(a) levels and genotype for the *IL6R* variant rs2228145 (UKB IL-6 subset, n = 44,233). Participant characteristics were summarized according to strata of the rs2228145 variant in the *IL6R* gene. Continuous variables were compared using the Kruskal-Wallis test, and categorical variables were compared using Pearson’s chi-square test. To evaluate the trends in lipid and laboratory parameters across groups of wild type (WT), heterozygous, and homozygous for rs2228145, Jonckheere–Terpstra trend tests were used to compare continuous variables and Cochran-Mantel-Haenszel linear trend tests were performed for categorical variables. Continuous data are presented as median (25th-75th percentiles), while categorical data are presented as count (percentage). Correlation of continuous variables was determined using Spearman's correlation coefficient (rho). In all tests, a *P* value <0.05 was considered statistically significant. All statistical analyses were conducted using R version 4.5.1 (R Core Team, 2025).

### MR study of IL-6R signaling on Lp(a)

We performed 2-sample inverse-variance weighted MR, sensitivity analysis with robust methods (simple median, weighted median, MR-Egger and PR-PRESSO), assessed the presence of influential SNVs based on leave-one-out estimates and performed colocalization analysis to assess causality of IL-6R signaling for Lp(a) concentrations using the MendelianRandomization (version 0.10.0),^52^^,^^53^ MRPRESSO (version 1.0),^54^ and coloc (version 5.2.3)^55^ packages for R. Importantly, while MR-Egger relaxes the exclusion restriction assumption, it assumes that an instrumental variable’s association with the exposure must be independent of its direct effects upon the outcome (InSIDE assumption).

We used 24 SNVs in the *IL6R* locus (Supplemental Table 2) separately as instrumental variables. Due to high heterogeneity, we also performed sensitivity analyses including only the 7 SNVs that are located within the *IL6R* gene. The association estimates with the exposure (natural log-transformed CRP) were taken from a meta-GWAS in 204,402 participants of European ancestry in the Cohorts for Heart and Aging Research in Genomic Epidemiology Consortium^56^ adjusted for age, sex, and population substructure (details in the Supplemental Methods). To estimate the effect of the instrumental variables on Lp(a) concentrations, we performed a GWAS on Lp(a) concentrations (rank inverse normally transformed) in White UKB participants (428,868) using the nf-gwas pipeline (version 1.0.9).^57^ The GWAS was adjusted for age, sex, genotype batch, and the first 30 Principal Components (PCs).

Colocalization analysis was performed as a sensitivity analysis for MR^58^ in the *IL6R* locus to compare the genetic association signal of CRP as a proxy for IL-6R signaling and Lp(a) (details in the Supplemental Methods). The posterior probability for the same causal variant (H4) is reported.

To assess the joint effect of IL-6R signaling and genetic variation in the *LPA* gene on absolute Lp(a) concentrations, we used the *IL6R* and *LPA* genetic score as explaining variables in 428,407 White individuals of the UKB.^59^ We performed quantile regression on Lp(a) concentration including an interaction term between the 2 scores adjusted for age, sex, genotyping batch, kinship, and the first 10 PCs.

To assess with a reverse MR whether Lp(a) concentrations causally affect IL-6 concentrations, we used the 14 SNVs in the *LPA* locus (Supplemental Table 4) separately as instrumental variables. The association estimates with the exposure [inverse normally transformed Lp(a)] were taken from a meta-GWAS in 13,781 European individuals with no overlap with UKB adjusted for age and sex^49^ (details in the Supplemental Methods). The association of the instrumental variables with IL-6 (rank inverse normally transformed protein levels) was derived from a GWAS in 33,657 White UKB participants adjusted for age, age^2^, sex, age × sex, age^2^ × sex, batch, UKB center, UKB genetic array, time between blood sampling and measurement, and the first 20 PCs.^42^ As a positive control, MR of coronary heart disease was performed with the same instrumental variables. The association of the instrumental variables with coronary heart disease was derived from a GWAS in 361,194 White UKB participants adjusted for age, age^2^, inferred sex, age x inferred sex, age^2^ x inferred sex, and the first 20 PCs, and accessed via openGWAS (ukb-d-I9_CHD) database API.^60^^,^^61^

Model results are presented as the regression coefficient (β) with 95% confidence interval (CI) and corresponding *P* values. Heterogeneity was assessed using Cochran’s Q, pleiotropy using the MR-Egger intercept, and the MR-PRESSO global test.

### Regression models

The association of IL-6 plasma levels and *IL6R* variant rs2228145 with Lp(a) concentrations was tested using quantile regression (R package “quantreg,” version 6.1, function rq). IL-6 levels were tested in the UKB IL-6 subset. The analysis was also stratified by number of rs2228145 functional copies (covariates = age, sex, estimated glomerular filtration rate, body mass index, hypertension, statins, diabetes, low-density lipoprotein cholesterol, high-density lipoprotein cholesterol, smoking status, and *LPA* score; n = 38,101). Rs2228145 was tested in the overall UKB cohort (covariates = age, sex; n = 428,407) and in the UKB IL-6 subset (covariates: age, sex, IL-6 levels; n = 44,233). We used a multivariable linear regression model on inverse normally transformed Lp(a) (R package “stats,” version 4.5.1, function lm) adjusted for the same confounders as the quantile regression and proportional marginal variance decomposition to evaluate the proportion of Lp(a) variance explained by IL-6 levels. The proportional marginal variance decomposition metric was calculated using R package “relaimpo” (version 2.2.7), with 1000 bootstrapping runs to calculate the 95% CIs. This approach decomposes the total variance explained (R^2^) into non-negative contributions by weighted averaging of sequential R^2^ values for all individual covariates that sum to the total R^2^ of the model.^62^

### Factorial MR of the combined effect of down-regulation of IL-6R signaling and Lp(a) on CVD

This analysis was performed in White UKB participants with *IL6R* and *LPA* genetic score, age, sex, kinship, genotyping batch, and PCs available (n = 458,181). Cox proportional hazards regression with age as timescale adjusted for sex, kinship, genotyping batch, and the first 10 PCs was used to estimate the association of the *IL-6R* and *LPA* score with CVD. Because we are using genetic instruments to investigate associations with the outcome, we use age as timescale, as previously done.^63^^,^^64^ Results are presented as HR with 95% CI and corresponding *P* values. Quantile regression to the median was used to estimate the association of the 2 scores with Lp(a) and CRP concentrations adjusting for the same covariates. Results are presented as the regression coefficient (β) with 95% CI. Three models were used. First, to mimic a factorial randomized trial of IL-6R and Lp(a) inhibitors and ensuring equally-sized groups, the cohort was split into 4 groups based on median score cutoffs: both scores above median genetically mimics the placebo group, *IL6R* score below median genetically mimics treatment with IL-6R inhibitors, *LPA* score below median genetically mimics treatment with Lp(a) inhibitors, and both scores below median genetically mimics treatment with both inhibitors.^43^^,^^59^^,^^65^ Second, the 2 continuous genetic scores were jointly included in regression models with an interaction term to check for multiplicative effects on CVD (estimates scaled to a 1-SD decrease).^59^ Third, the 2 continuous genetic scores were jointly included in regression models without an interaction term, also scaled to a 1-SD decrease to mimic down-regulation.

## Results

### MR study supports causal role of IL-6R signaling in Lp(a) concentrations

We assessed the association between genetically proxied IL-6R signaling and Lp(a) concentration leveraging 24 *IL6R*-locus SNVs as instrumental variables within an MR framework in European cohorts. Two-sample inverse-variance weighted MR indicated a significant causal association of IL-6R signaling with Lp(a) concentrations (β = 0.06; 95% CI: 0.04-0.09). Despite the significant heterogeneity (Cochran’s Q = 36.90; *P =* 0.033) (Supplemental Figure 1A), sensitivity analyses with robust methods (simple median, weighted median, MR-Egger, and MR-PRESSO) consistently supported a significant causal association with similar estimates (Supplemental Table 5). The MR-Egger intercept and the global MR-PRESSO test were not significant *(P =* 0.62 and *P >* 0.99, respectively), indicating absence of pleiotropy. Leave-1-out estimates did not reveal single influential SNVs (Supplemental Figure 2). Sensitivity analysis with 7 SNVs that were located within the *IL6R* gene was also significant (β = 0.09, 95% CI: 0.07-0.12) with no indication of heterogeneity (Cochran’s Q = 4.51; *P =* 0.61) (Supplemental Figure 1B). In addition, colocalization analysis in the *IL6R* locus indicated a high posterior probability for the same causal variant (H4 = 0.975) of the genetic association signal of CRP as a proxy for IL-6R signaling and Lp(a) concentrations.

Lp(a) concentrations are mainly determined by the *LPA* gene.^66^ We assessed the joint association of genetically proxied IL-6R signaling and genetic variation in the *LPA* gene on absolute Lp(a) concentrations, using the *IL6R* and *LPA* genetic score as explaining variables in 428,407 White UKB individuals. The 2 scores were not significantly correlated (rho < 0.01; *P =* 0.81). Adjusted quantile regression on Lp(a) concentration showed that both the *IL6R* score (β = 0.07 nmol/L per SD increase; 95% CI: 0.02-0.11; *P =* 0.003) and the *LPA* score (β = 55.98 nmol/L per SD increase; 95% CI: 55.81-56.15; *P <* 0.001) were significantly associated with Lp(a) concentrations. Additionally, there was a significant interaction between the 2 scores (β = 0.36 nmol/L per SD increase; 95% CI: 0.19-0.52; *P <* 0.001) suggesting a stronger effect of increased genetic IL-6R signaling on Lp(a) concentrations at higher values of the *LPA* score.

Reverse MR using SNVs in the *LPA* region as instrumental variables for Lp(a) levels showed no evidence of a causal linear effect of Lp(a) concentrations on plasma IL-6 concentrations (Supplemental Figure 3A, Supplemental Table 6). In the positive control analysis, the same instruments showed a causal association with coronary heart disease (Supplemental Figure 3B).

### The *IL6R* LOF VARIANT rs2228145 affects the association between IL-6 circulating levels and Lp(a) concentrations

Having inferred the directionality from IL-6R signaling toward Lp(a) concentrations, we investigated the magnitude of its effect at population level.

Our overall UKB cohort consisted of 471,607 White participants. Of them, 44,233 had available data on IL-6 and Lp(a) levels and genotypes for the LOF *IL6R* rs2228145 variant (UKB IL-6 subset). Supplemental Table 7 summarizes baseline characteristics of these participants.

In both analysis cohorts, ≈48% and ≈17% of individuals were heterozygous and homozygous for the rs2228145 minor allele, respectively. Table 1 and Supplemental Table 8 show the baseline characteristics stratified by the rs2228145 genotype of the UKB IL-6 subset and the entire UKB cohort, respectively. Trends in lipid profiles and laboratory parameters across the different strata of rs2228145-C genotype are shown in Table 2 and Supplemental Table 9. The minor allele (rs2228145-C) increases proteolytic cleavage of the membrane-bound IL-6R, thereby down-regulating the classical signaling pathway of IL-6.^45^^,^^46^ Concordantly, there was a significant decrease in CRP concentrations across the 3 groups, with homozygous individuals having the lowest CRP concentration compared with WT (1.17 mg/L vs 1.48 mg/L; *P*_trend_ < 0.001) (Table 2). In line with previous findings,^16^^,^^30^ circulating IL-6 levels significantly increased across the strata, with homozygotes for the rs2228145 minor allele displaying the highest IL-6 levels (*P*_trend_ < 0.001). Interestingly, this was accompanied by a trend toward lower Lp(a) concentrations with presence of the *IL6R* variant, which reached significance when tested in the overall UKB cohort (*P*_trend_ = 0.004) (Supplemental Table 9)**.** Based on these observations, we addressed the interplay between the 2 primary determinants of the IL-6R signaling, IL-6 plasma levels, and IL-6R functional state.

**Table 1:** Baseline Characteristics of Participants in the UK Biobank Subset^a^ (N = 44,233) Stratified Based on SNV in *IL6R* (rs2228145)

	Wild Type (A/A)	Heterozygous (A/C)	Homozygous (C/C)	*P* Value
Sample size^b^	15,571 (35)	21,257 (48)	7,405 (17)	
Demographics				
Men	7,149 (45.9)	9,862 (46.4)	3,390 (45.8)	0.53
Age, y	59.00 (51.00-64.00)	59.00 (51.00-64.00)	59.00 (51.00-64.00)	0.85
Body mass index, kg/m^2^	26.78 (24.16-29.92)	26.75 (24.17-29.86)	26.70 (24.13-29.79)	0.50
Smoking status				0.35
Never	8,226 (52.8)	11,424 (53.7)	3,929 (53.1)	
Previous	5,661 (36.4)	7,587 (35.7)	2,649 (35.8)	
Current	1,634 (10.5)	2,182 (10.3)	802 (10.8)	
Baseline clinical conditions				
Hypertension	8,726 (56.1)	11,839 (55.7)	4,121 (55.7)	0.78
Diabetes	761 (5.1)	1,012 (5.0)	359 (5.0)	0.88
Cardiovascular disease	881 (5.7)	1,185 (5.6)	393 (5.3)	0.71
Autoimmune disease	624 (4.0)	792 (3.7)	272 (3.7)	0.30
Rheumatoid arthritis	180 (1.2)	211 (1.0)	73 (1.0)	0.27
Medication				
Statins	2,822 (18.1)	3,686 (17.3)	1,319 (17.8)	0.14

SNV = single-nucleotide variation.

Values are median (25th-75th percentiles) or n (%). Cardiovascular disease cases are prevalent cardiovascular disease events (as defined in Supplemental Table 1) that occurred before enrollment.

a Subset of individuals with available data on circulating interleukin (IL)-6, lipoprotein(a) and *IL-6R* variant (rs2228145).

b Sample size within each stratified group is expressed as count (percentage), where percentage represents the proportion relative to the UK Biobank subset (n = 44,233).

**Table 2:** Trends in Lipid Profiles and Laboratory Parameters in the UK Biobank Subset^a^ (N = 44,233) Stratified Based on SNV in *IL6R* (rs2228145)

	Wild Type (A/A)	Heterozygous (A/C)	Homozygous (C/C)	*P*_trend_ Value
Sample size^b^	15,571 (35)	21,257 (48)	7,405 (17)	
Lipid profile				
Total cholesterol, mmol/L	5.62 (4.86-6.41)	5.64 (4.88-6.42)	5.64 (4.86-6.43)	0.18
LDL cholesterol, mg/dL	134.98 (112.33-158.87)	135.06 (113.08-159.34)	135.60 (112.25-159.45)	0.27
HDL cholesterol, mmol/L	1.40 (1.17-1.68)	1.40 (1.17-1.68)	1.41 (1.16-1.68)	0.65
Triglyceride, mmol/L	1.49 (1.05-2.15)	1.49 (1.06-2.15)	1.49 (1.05-2.17)	0.42
Lp(a), nmol/L	19.19 (7.46-73.81)	18.60 (7.32-71.64)	18.37 (7.36-69.63)	0.059
Laboratory parameters				
eGFR	92.63 (82.54-99.73)	92.56 (82.21-99.70)	92.81 (82.72-99.77)	0.98
UACR, mg/g	9.22 (5.80-18.63)	9.60 (5.98-19.59)	9.29 (5.87-18.37)	0.44
CRP, mg/L	1.48 (0.72-3.02)	1.32 (0.65-2.76)	1.17 (0.58-2.40)	<0.001
Interleukin-6	−0.14 (−0.59 to 0.39)	0.00 (−0.43 to 0.55)	0.22 (−0.22 to 0.78)	<0.001

Values for categorical measures are presented as count (percentage) and for continuous data as median (25th-75th percentiles).

CRP = high-sensitivity C-reactive protein; eGFR = estimated glomerular filtration rate; HDL = high-density lipoprotein; LDL = low-density lipoprotein; SNV = single-nucleotide variation; UACR = urine albumin-creatinine ratio.

a Subset of individuals with available data on circulating interleukin (IL)-6, lipoprotein(a) [Lp(a)], and *IL-6R* variant (rs2228145).

b Sample size within each stratified group is expressed as count (percentage), where percentage represents the proportion relative to the UK Biobank subset (n = 44,233). Statistically significant *P* values are depicted in bold.

The rs2228145 minor allele significantly associated with a small reduction in median Lp(a) concentrations in both the overall UKB cohort (β = −0.25 nmol/L per rs2228145-C; 95% CI: −0.42 to −0.09; *P =* 0.003; n = 428,407) and in the UKB IL-6 subset (β = −0.55 nmol/L per rs2228145-C; 95% CI: −1.09 to −0.02; *P =* 0.043; n = 44,233). Importantly, the association was refined when adjusting for IL-6 plasma levels (β = −0.71 nmol/L per rs2228145-C; *P =* 0.009; n = 44,233) (Table 3).

**Table 3:** Quantile Regression of *IL6R* Loss Of Function Variant rs2228145 on Lipoprotein(a) Concentration

	n	SNV (Wild Type/Heterozygotes/Homozygotes)	Covariates	β (95% CI)^a^	*P* Value
UKB overall	428,407	149,964/206,128/72,315	Age and sex	−0.250 (−0.416 to −0.084)	0.003
UKB IL-6 subset	44,233	15,571/21,257/7,405	Age and sex	−0.552 (−1.086 to −0.018)	0.043
UKB IL-6 subset	44,233	15,571/21,257/7,405	Age, sex, and IL-6	−0.706 (−1.234 to −0.177)	0.009

IL = interleukin; SNV = single-nucleotide variation; UKB = UK Biobank; WT = wild type.

a β-estimates are given per loss-of-function allele. Each variable used in the models was available for all tested individuals.

Concordantly, IL-6 plasma levels were significantly associated with increased Lp(a) concentrations (β = +0.82 nmol/L; 95% CI: 0.59-1.05 nmol/L; *P <* 0.001; n = 38,101) (Table 4) and the β-estimate increased to +0.91 nmol/L (95% CI: 0.69-1.13 nmol/L) when adjusting for rs2228145 (P *<* 0.001; n = 38,101) (Table 4).

**Table 4:** Quantile Regression of IL-6 Plasma Levels on Lp(a) Concentration in the UK Biobank (UKB) IL-6 Subset

	β (95% CI)^a^	*P* Value	Variance of Lp(a) Explained by IL-6 in % (95% CI)^b^	n
UKB IL-6 subset	+0.819 (0.586-1.053)	<0.001	0.03 (0.01-0.06)	38,101
UKB IL-6 subset additionally adj for rs2228145	+0.910 (0.692-1.128)	<0.001	0.04 (0.02-0.06)	38,101
rs2228145 WT (A/A)	+1.065 (0.878-1.253)	<0.001	0.06 (0.02-0.13)	13,402
rs2228145 WT (A/A) or Heterozygous (A/C)	+1.029 (0.796-1.261)	<0.001	0.05 (0.02-0.08)	31,691
rs2228145 Homozygous (C/C)	+0.412 (−0.223 to 1.047)	0.17	0.00 (0.00-0.05)	6,410

All models were adjusted for age, sex, smoking status, hypertension, estimated glomerular filtration rate, body mass index, low-density lipoprotein cholesterol, high-density lipoprotein cholesterol, diabetes, statins, and *LPA* score. Each variable used in the models was available for all tested individuals. ^a^β-estimates are given per unit increase of IL-6 plasma levels in the UKB subset. ^b^R^2^ in percent of IL-6 calculated by proportional marginal variance decomposition. It estimates the variance of Lp(a) concentrations explained by IL-6 in each model.

Abbreviations as in Tables 2 and 3.

**Figure 1:**
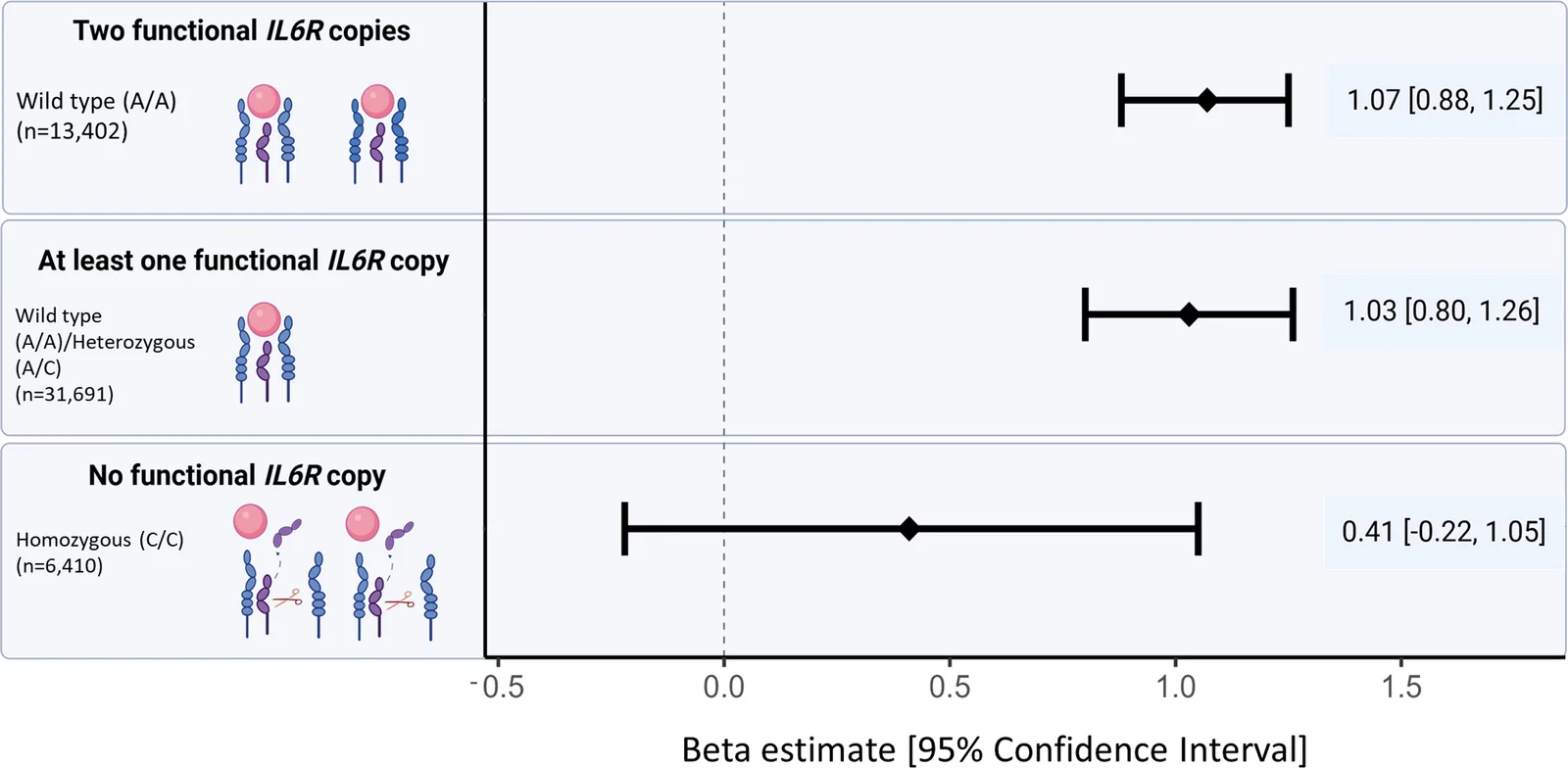
Association of Circulating IL-6 and Lp(a) Concentrations-Stratified by rs2228145 Genotype Forest plot showing regression coefficient (β) from quantile regression in an analysis in UK Biobank interleukin (IL)-6 subset (n = 38,101) stratified by rs2228145 genotype. All models were adjusted for age, sex, smoking status, hypertension, estimated glomerular filtration rate, body mass index, low-density lipoprotein cholesterol, high-density lipoprotein cholesterol, diabetes, statins, and *LPA* score. From top to bottom: association shown in subsets of the following: 1) individuals with 2 functional copies of rs2228145 (A/A; *P* < 0.001); 2) at least 1 functional copy (A/A)/(A/C), *P <* 0.001; and 3) no functional copy (C/C); *P =* 0.17. Reference at β = 0 (dashed line). Figure created using Biorender.com.

Because IL-6R is downstream of IL-6 plasma levels in the signaling cascade, we stratified by rs2228145. The association between IL-6 plasma levels and Lp(a) concentrations was modulated by the number of functional WT copies of rs2228145 (Figure 1 and Table 4). The β-estimates ranged from +1.03 to +1.07 nmol/L in the groups carrying at least 1 functional WT allele (n = 31,691; *P* < 0.001) and 2 functional WT alleles (n = 13,402; *P* < 0.001), respectively. When both *IL6R* alleles carried the LOF variant (n = 6,410), the β-estimate was reduced by 2.6-fold (β = +0.41 nmol/L) and the association was not significant *(P =* 0.17). Supporting the overall minor contribution of IL-6R signaling to Lp(a) concentrations, IL-6 levels explained 0.03% of the Lp(a) variance in the UKB IL-6 subset (Table 4). The variance explained increased to 0.04% when adjusting for the SNV rs2228145. Consistent with the biological relevance of the IL-6R functional state, the contribution of IL-6 levels to Lp(a) concentration was amplified in individuals with 2 functional WT copies of rs2228145 (analysis stratified by rs2228145 functional copies), explaining 0.06% of the Lp(a) variance (Table 4).

### Genetic down-regulation of IL-6R signaling and Lp(a) independently protect against CVD

**Figure 2:**
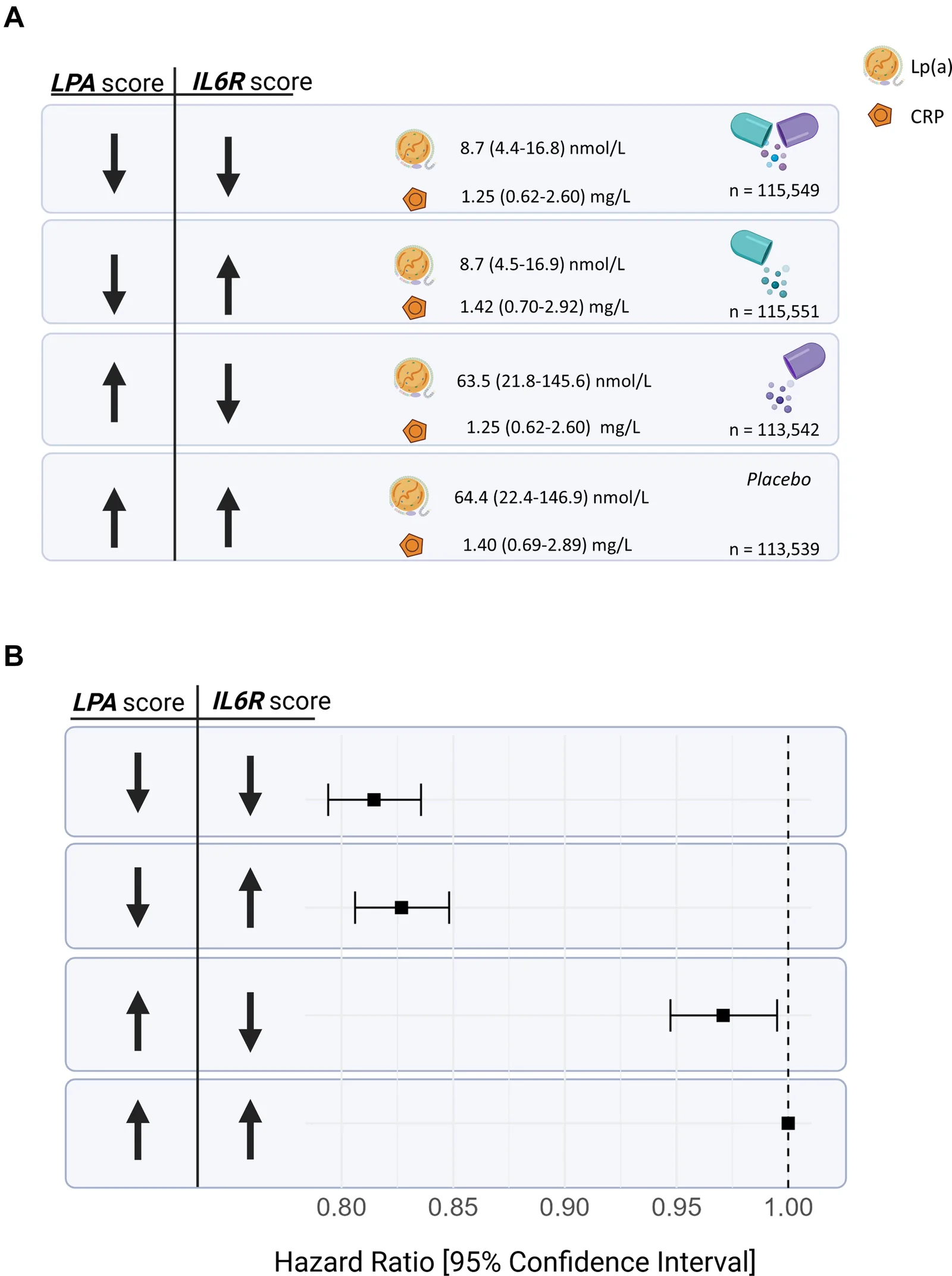
Independent Effect of *LPA* and *IL6R* Scores on CVD Risk (A) Schematic diagram of the setup used for factorial Mendelian randomization. The cohort was separated into 4 groups based on median genetic scores: 1) both scores > median mimicking the placebo group; 2) *IL6R* score≤median mimicking IL-6R signaling inhibition; 3) *LPA* score ≤ median mimicking Lp(a) reduction; and 4) both scores ≤ median mimicking parallel down-regulation. Lp(a) in nmol/L and CRP in mg/L. Data are presented as median (25th-75th percentiles). (B) Forest plot of HR and 95% CIs for CVD in UKB stratified by *IL6R* and *LPA* scores with both scores > median (mimicking the placebo group in a clinical trial) set as reference (dashed line). HR from a Cox proportional hazard model with age as timescale additionally adjusted for sex, kinship, genotyping batch, and the first 10 genetic principal components. Figure created using Biorender.com.

To test the joint effects and interaction of lifelong genetic down-regulation of IL-6R signaling (proxied by CRP) and Lp(a) concentrations on CVD, we used the *IL6R* and the *LPA* score (derived from European cohorts), respectively. Analyses were performed in 458,181 White individuals in the UKB with relevant variables available. The cohort was separated into 4 groups based on median score levels (Figure 2). The baseline characteristics of the 4 groups are presented in Supplemental Table 10. Taking as reference the group where both *LPA* and *IL6R* scores were above the median, the Lp(a) concentrations were lowered by −55.54, −1.02, and −55.55 nmol/L in individuals with *LPA* score only, *IL6R* score only, and both scores below the median, respectively (Table 5). Accordingly, CRP levels changed by 0.02, −0.15, and −0.14 mg/L in the respective groups (Table 5).

**Table 5:** Joint analysis of *IL6R* and *LPA* Score Decrease on Cardiovascular Disease

	n	Effect on Lp(a) β (95% CI)^a^	Effect on CRP β (95% CI)^b^	No. of Events	HR (95% CI)^c^	*P* Value of the Cox Model
Grouped analysis						
Both scores ≤ median	115,549	−55.55 (−56.40 to −54.70)	−0.14 (−0.16 to −0.13)	10,805	0.81 (0.79-0.84)	<0.001
*LPA* score ≤ median	115,551	−55.54 (−56.39 to −54.69)	0.02 (0.01 to 0.04)	10,876	0.83 (0.81-0.85)	<0.001
*IL6R* score ≤ median	113,542	−1.02 (−2.20 to 0.16)	−0.15 (−0.17 to −0.14)	12,458	0.97 (0.95-1.00)	0.018
Both scores > median	113,539	Reference	Reference	12,773	Reference	Reference
Continuous analysis additive model						
*IL6R* score (1-SD decrease)	458,181	−0.14 (−0.19 to −0.09)	−0.10 (−0.10 to −0.09)	46,912	0.99 (0.98-1.00)	0.019
*LPA* score (1-SD decrease)	458,181	−55.96 (−56.12 to −55.79)	0.00 (−0.00 to 0.01)	46,912	0.88 (0.87-0.89)	<0.001
Continuous analysis interaction model						
*IL6R* score (1-SD decrease)	458 to 181	−0.32 (−0.44 to −0.19)	−0.10 (−0.10 to −0.09)	46,912	0.99 (0.98-1.00)	0.027
*LPA* score (1-SD decrease)	458 to 181	−55.98 (−56.15 to −55.82)	0.00 (−0.00 to 0.01)	46,912	0.88 (0.87-0.89)	<0.001
Interaction (1-SD decrease)	458 to 181	0.36 (0.19 to 0.52)	−0.00 (−0.01 to 0.00)	46,912	1.00 (1.00-1.01)	0.28

a Effect on Lp(a): based on a quantile regression to the median adjusted for age at enrollment, sex, kinship, genotyping batch, and the first 10 genetic principal components.

b Effect on C-reactive protein (CRP): based on a quantile regression to the median additionally adjusted for age at enrollment, sex, kinship, genotyping batch, and the first 10 genetic principal components.

c HR of a Cox proportional hazard model with age as timescale additionally adjusted for sex, kinship, genotyping batch, and the first 10 genetic principal components.

Genetically lower levels of Lp(a) (HR: 0.83; 95% CI: 0.81-0.85; *P <* 0.001) and down-regulation of IL-6R signaling (HR: 0.97; 95% CI: 0.95-1.00; *P =* 0.018) were independently associated with a reduced risk of CVD (Table 5, Figure 2). Individuals with genetically lower levels of both Lp(a) and IL-6R signaling had a 19% lower risk of CVD (HR: 0.81; 95% CI: 0.79-0.85; *P <* 0.001). The interaction term between continuous genetic scores was not statistically significant *(P =* 0.28), providing no evidence for departure from additivity on the log-risk scale (Table 5).

## Discussion

In the work at hand, 2-sample MR analyses supported the causal role of IL-6R signaling in increasing Lp(a) concentrations. Notably, the positive association between circulating levels of IL-6 and Lp(a) was modulated by the functional state of the IL-6R, suggesting that neglecting the receptor confounds the association between IL-6 plasma levels and Lp(a) concentrations. We also show that, despite consistency with causality, the effect of IL-6R signaling on Lp(a) concentration in the general population is marginal. In line, our factorial MR showed that genetic down-regulation of IL-6R signaling and Lp(a) confer independent CVD risk reduction.

Both IL-6^8^^,^^9^ and Lp(a)^10^^,^^59^ inhibitors are in phase 3 clinical trials for CVD. Growing evidence supports a bidirectional relationship between these nontraditional CVD risk factors.^67^ On one hand, the presence of IL-6 response elements in the *LPA* promoter has historically suggested a direct link between IL-6 and Lp(a).^67^ On the other hand, Lp(a)-induced inflammatory effects have been attributed to different particle components,^67^ including oxidized phospholipids carried by Lp(a).^68^^,^^69^ A recent genetic study showed that IL-6 inhibition reduces Lp(a) concentrations,^28^ and in clinical trials, medications blocking either IL-6^8^ or its receptor resulted in Lp(a) reduction by 16% to 41%.^29^^,^^70^^,^^71^ Hence, we focused on the IL-6R activity.

Our 2-sample MR supported a causal role of IL-6R signaling in Lp(a) concentrations. These findings are also consistent with mechanistic evidence showing that IL-6R signaling plays a direct regulatory role in Lp(a) biology.^26^^,^^27^^,^^67^

We tested reverse causality of Lp(a) concentrations in IL-6 levels using *LPA* genetic instruments of established validity (high F statistic and replicated association with coronary heart disease^19^). Our analysis showed no indication of a linear causal effect of Lp(a) concentrations on systemic IL-6 concentrations in the general population. This is in line with an MR study finding no evidence of a causal effect of Lp(a) on low-grade inflammation proxied by CRP.^72^ On the contrary, a mechanistic study showed that monocytes incubated with high Lp(a) concentration display an OxPLs-driven increase in IL-6 after a stimulation step.^73^ However, it must be considered that the stimulation step is not translatable to the general population, and our genetic instruments are designed to capture Lp(a) concentration and not oxidized phospholipids.^73^

We further aimed to disentangle the contributions of IL-6 plasma levels and IL-6R functional state in increasing Lp(a) at epidemiological level. In UKB, 17% of the individuals are homozygous for the LOF *IL6R* variant rs2228145.^16^^,^^17^ This variant increases proteolytic cleavage of the membrane-bound IL-6R, reducing classical IL-6R signaling.^45^^,^^46^ In our cohort, we observed a significant trend for increasing IL-6 plasma levels (*P*_*trend*_ < 0.001**)** and decreasing Lp(a) concentrations (*P*_*trend*_ = 0.004) across the 3 rs2228145 genotype groups, with homozygotes for the minor allele displaying the highest IL-6 and the lowest Lp(a) concentrations. Although the increase in IL-6 levels is well-known,^16^^,^^30^ this is not in line with the hypothesis that higher IL-6 levels cause higher Lp(a) concentrations. This discrepancy suggests that the functional state of IL-6R is biologically a limiting factor in the association between IL-6 plasma levels and the Lp(a) concentration that should be considered in epidemiological studies.

Consistently, the association between IL-6 plasma levels and Lp(a) concentrations was modulated by the number of functional IL-6R copies as determined by rs2228145, with the point-estimate increasing by 2.6-fold in WT for the functional allele vs homozygous for the minor allele. Thus, IL-6R functional state acts as a statistical confounder in the association between circulating levels of IL-6 and Lp(a). In individuals with 2 minor alleles of rs2228145, the association of IL-6 plasma levels and Lp(a) concentrations did not reach significance. This is in line with the hypothesis that at least 1 functional copy of the IL-6R is needed to activate the intracellular signaling cascade influencing *LPA* transcription. However, this finding needs replication in larger cohorts.

IL-6 signals by binding either to membrane-bound IL-6R (classical signaling), binding to soluble IL-6R (trans-signaling), or being presented by dendritic cells to T cells via membrane IL-6R *(transpresentation)*.^45^ Because the rs2228145 (Asp358Ala) variant primarily impairs the classical signaling pathway of IL-6,^46^ while increasing soluble IL-6R,^45^^,^^74^ we speculate that, in the general population, IL-6 levels modulate the Lp(a) concentrations mainly through the classical signaling pathway. Biologically, this is highly plausible because *LPA* is exclusively expressed in the liver,^75^ and hepatocytes express IL-6R on their membranes and are thus responsive to the classical signaling pathway.^45^ Nevertheless, statistical analyses cannot conclusively identify the specific IL-6 receptor signaling pathway involved. Future experimental studies addressing the signaling pathways are warranted.

Overall, the magnitude of IL-6R signaling effect on Lp(a) concentrations in the general population was marginal, explaining 0.04% and 0.03% of the variance with and without adjustment for rs2228145, respectively. This modest impact and the high frequency of individuals homozygous for the LOF rs2228145 (∼17%) may provide an explanation for the undetected positive association between IL-6 and Lp(a) in previous studies.^31^^,^^76^ Additionally, the small influence of IL-6 levels on Lp(a) is consistent with the major role played by a complex pattern of sequence variants within the *LPA* gene.^66^ However, fluctuations in Lp(a) variance can arise from other factors, such as inflammation and chronic kidney disease.^19^ Concordantly, clinical trials showing prominent reduction of Lp(a) concentrations after down-regulating IL-6R signaling were exclusively conducted in rheumatoid arthritis and chronic kidney disease populations.^29^ The elevated inflammatory markers in these populations might explain the pronounced reduction in Lp(a) after treatment with IL-6R signaling inhibitors.^29^

Interestingly, we also detected a significant statistical interaction between the *IL6R* score and the *LPA* score. This translates into a larger effect of the IL-6R signaling as the *LPA* score increases. Indeed, IL-6R signaling increases the *LPA* gene transcription, while functional sequence variants within *LPA* have been reported to affect splicing^63^^,^^77^ and post-translational processing.^78^^,^^79^ Thus, it is biologically plausible that the absolute impact of the IL-6R signaling is amplified with increasing *LPA* score [ie, higher Lp(a) concentrations]. This points toward the consideration that these patients might benefit more of IL-6R signaling inhibitors than individuals with low Lp(a). In line, CVD clinical trial for low-dose colchicine 2, which indirectly attenuates IL-6 and its downstream mediator CRP showed a larger protective effect against CVD in patients with high versus low Lp(a).^32^ Further investigations are required.

### Clinical implications

It is currently highly debated whether the connection between the IL-6R signaling cascade and Lp(a) may impact patient selection for CVD treatment with inhibitors for IL-6R signaling and Lp(a).^31^, ^32^, ^33^

Factorial MR of genetic down-regulation of IL-6R signaling and Lp(a) concentrations showed that they are independently associated with CVD risk reduction. Using the *IL6R* and *LPA* scores on the continuous scale showed similar results with no indication of interaction on the log-risk scale. Some observational studies have recently reported that IL-6^31^^,^^32^ and CRP^36^ modify the association of Lp(a) with CVD risk, with the association only being present in individuals with increased inflammatory markers. However, Lp(a) has also been reported to be associated with CVD independently of CRP^39^^,^^80^^,^^81^ and IL-6 levels.^33^ Our analysis based on lifelong genetic down-regulation indicates that there is an independent protective effect of down-regulated IL-6R signaling and Lp(a) on CVD risk. This may help refining patient selection for inhibitors for IL-6R signaling and Lp(a) and establish a basis for identifying complementary opportunities.

### Study limitations

MR genetic proxies in the *IL6R* and *LPA* loci were derived by studies in Europeans.^47^, ^48^, ^49^^,^^56^^,^^82^ Thus, we restricted our analyses to UKB participants of White ancestry. Genetic instruments that are either ancestry-specific or robust across ancestries are urged to translate these investigations to other populations. The genetic instruments were used to approximate the effects of IL-6R inhibition and Lp(a) inhibition. However, it is important to acknowledge that these proxies may not fully replicate the pharmacological effects of the corresponding therapies and represent lifelong changes in the proxied trait. Moreover, several analyses could only be performed in the smaller Pharma Proteomics Project subset of UKB, where IL-6 plasma levels are available levels as unitless inverse-rank normalized protein expression.^42^ The subset may introduce selection bias, as explained in Sun et al.^42^ However, it should be noted that 91% of the proteomic analyses in UKB were performed on a random sample with characteristics similar to those of the overall UKB population.^42^

## Conclusions

The IL-6R signaling cascade appears to causally influence Lp(a) concentrations. Importantly, the functional state of the IL-6R is a biological limiting factor in the signaling cascade, and neglecting it can confound the association between IL-6 plasma levels and Lp(a) concentrations. Despite indications for causality, this pathway explains only a minor portion of the Lp(a) variance in the general population. In line, factorial MR suggests that inhibiting both IL-6R signaling and Lp(a) concentrations likely provides independent reduction of CVD risk.

Perspectives **COMPETENCY IN MEDICAL KNOWLEDGE:** CVD arises from a complex interplay of established and emerging risk factors. Disentangling their individual contributions is essential for designing health care packages that ensure timely detection and effective management. Our MR analyses indicate independent causal effects of IL-6R signaling and Lp(a) concentrations, highlighting their clinical relevance. Notably, medications targeting these 2 emerging risk factors are currently in phase 3 clinical trials for CVD. Until such therapies become widely available, our findings underscore the importance of increasing awareness of both risk factors to support personalized strategies for managing traditional cardiovascular risk profiles.

**TRANSLATIONAL OUTLOOK:** With phase 3 trials targeting IL-6R signaling and Lp(a) nearing completion, our results may help refine patient selection and guide the integration of emerging treatments into personalized prevention approaches. Our findings support exploring their combination in future clinical trials. This may enhance risk reduction beyond current therapies, especially in high-risk populations, such as those with elevated inflammatory burden.

## Funding Support and Author Disclosures

This work is supported by an Lp(a) Research Grant from the Austrian Atherosclerosis Society to Dr Schachtl-Riess. This research was funded in whole or in part by the Austrian Science Fund (FWF) 10.55776/W1253. For open access purposes, the author has applied a CC BY public copyright license to any author accepted manuscript version arising from this submission. Dr Coassin has received honoraria from Novartis AG (Basel, CH) and Silence Therapeutics PLC (London, UK) for consultancy on Lipoprotein(a) genetics. Dr Kronenberg has received consulting or lecture fees from Novartis, Amgen, Silence Therapeutics, AstraZeneca, General Atlantics, Medscape, and Roche Diagnostics. All other authors have reported that they have no relationships relevant to the contents of this paper to disclose.
