# Supplementary material for: Mendelian Randomization Reveals Interleukin-6 Receptor-Lipoprotein(a) Interplay With Independent Cardiovascular Risk Reductions
Source: JACC Basic Transl Sci. 2026 May 8;11(8):101564. doi: 10.1016/j.jacbts.2026.101564 (PMC13523421; doi:10.1016/j.jacbts.2026.101564)
Supplement: Supplementary file 1 [file mmc1.docx]

**Supplemental Materials to:**

**^Mendelian Randomization Reveals Interleukin-6 Receptor-Lipoprotein(a) Interplay with Independent Cardiovascular Risk Reductions^**

Running Title: Causal IL-6R–Lp(a) Interplay with Independent Cardiovascular Risk

Azin Kheirkhah, PhDª⁺, Silvia Di Maio, PhDª⁺, Stefan Coassin, PhDª, Sebastian Schönherr, PhDª, Claudia Lamina, PhDª, Lukas Forer, PhDª, Florian Kronenberg, MDª, Johanna F. Schachtl-Riess, PhDª*

⁺Azin Kheirkhah and Silvia Di Maio contributed equally to this work

ªInstitute of Genetic Epidemiology, Medical University of Innsbruck, Innsbruck, Austria

*corresponding author

Correspondence to:

Johanna F. Schachtl-Riess, PhD

Institute of Genetic Epidemiology

Medical University of Innsbruck

Schöpfstrasse 3-6020 Innsbruck, AUSTRIA

Phone: (+43) 512 9003-70569

Email: [johanna.schachtl-riess@i-med.ac.at](mailto:johanna.schachtl-riess@i-med.ac.at)

Table of Contents

[Supplemental Methods	3](#_Toc221871813)

[The UK Biobank Study	3](#_Toc221871814)

[Genome-wide genetic data in UK Biobank	3](#_Toc221871815)

[Measurement of Lp(a) concentrations	3](#_Toc221871816)

[Plasma proteomics profiling	3](#_Toc221871817)

[Definition of CVD, comorbidities and covariates	4](#_Toc221871818)

[Instrumental variables for IL-6R signaling	4](#_Toc221871819)

[Instrumental variables for Lp(a) concentrations	5](#_Toc221871820)

[Colocalization analysis	5](#_Toc221871821)

[Supplemental Figures	7](#_Toc221871822)

[Supplemental Figure 1. IL-6R signaling is causally associated with Lp(a).	7](#_Toc221871823)

[Supplemental Figure 2. Leave-one-out estimates of the two-sample MR on Lp(a).	8](#_Toc221871824)

[Supplemental Figure 3. Lp(a) is not causally associated with IL-6 plasma levels but with CHD.	9](#_Toc221871825)

[Supplemental Tables	10](#_Toc221871826)

[Supplemental Table 1. Definition of cardiovascular disease and comorbidities in UK Biobank (UKB)	10](#_Toc221871827)

[Supplemental Table 2. Instrumental variables for IL-6R signaling (proxied by natural log transformed CRP concentrations) in the *IL6R* locus.	11](#_Toc221871828)

[Supplemental Table 4. Instrumental variables for Lp(a) concentrations in the *LPA* locus.	13](#_Toc221871829)

[Supplemental Table 5. Results of the two-sample MR on Lp(a) concentrations using 24 SNPs in the IL6R region as instrumental variables.	14](#_Toc221871830)

[Supplemental Table 6. Results of the two-sample MR on IL-6 plasma concentrations using 14 SNPs in the *LPA* region as instrumental variables.	15](#_Toc221871831)

[Supplemental Table 7. Baseline characteristics of the UK Biobank cohort and the subset of participants included in the observational analysis.	16](#_Toc221871832)

[Supplemental Table 10. Baseline table stratified into four groups based on median IL-6R and LPA score groups.	19](#_Toc221871833)

[References	20](#_Toc221871834)

# Supplemental Methods

## The UK Biobank Study

UK Biobank (UKB) is a prospective cohort study with >500,000 participants recruited between 2006-2010 in accordance with the Declaration of Helsinki (age at enrollment 40-69 years). The study was approved by the North West Multi-centre Research Ethics Committee. In this work, we selected participants of White ancestry based on the DataField 21000 (entries “British”, “Irish” or “Any other White background”), who did not withdraw as of August 2025 (application number 62905).

## Genome-wide genetic data in UK Biobank

Genome-wide genetic data were generated as described elsewhere^1^. In brief, genotyping data were generated using two similar genotyping arrays (Applied Biosystems UK BiLEVE Axiom Array and UK Biobank Axiom Array)^1^. After quality control, genotypes were available for 488,377 UKB participants. Imputed data were first generated using the Haplotype Reference Consortium (HRC)^2^ as main imputation reference and then combined with data imputed using the merged UK10K and 1000 Genome Phase 3 reference panels^3^. These data were available in 487,442 individuals^1^.

## Measurement of Lp(a) concentrations

Lp(a) concentrations were measured at recruitment with an immunoturbidimetric Denka Seiken assay (Randox Laboratories; Crumlin, County Antrim, United Kingdom using a Beckman Coulter AU5800 Platform), with a reportable range of 3.8-189 nmol/L. In total, 46,836 participants had Lp(a) levels below and 32,953 participants had levels above the reportable range. We obtained values outside the reportable range through a separate request. Further details are provided elsewhere^4^.

## Plasma proteomics profiling

As part of the UK Biobank Pharma Proteomics Project (UKB-PPP) initiative, 54,219 UK Biobank participants were collected for  proteomic profiling on blood plasma samples using the antibody-based Olink Explore 3072 proximity extension assay (PEA), as described elsewhere^5^. Of them, 6,376 samples were pre-selected by the Consortium members, 1,269 were picked based on the participation in the COVID-19 repeat-imaging study and the remaining 46,595 participants were randomly selected at baseline^5^. In total, 2.923 unique proteins were measured across eight protein panels. Quality control steps are described by Sun and colleagues^5^.

## Definition of CVD, comorbidities and covariates

We defined CVD considering either the first reported occurrence (as defined by UKB) falling under the International Classification of Disease version 10 (ICD10) codes I21-I25, I63, I64, I71 or the first reported CVD-related operative procedure (self-reported or hospital inpatients data). Since we are using genetic instruments to investigate associations with the outcome, we use age as timescale, as previously done^6,7^. In total, 48,780 participants were classified as CVD incident cases in the timeframe spanning from birth to censoring date (1st of January 2020). Thereof, 23,330 occurred before enrollment (classified as prevalent CVD cases, . Within the UKB IL-6 subset there were 5,210 CVD cases (2,459 before enrollment). **Supplemental Table 1** includes detailed information about the UKB DataFields and codes selected to define CVD and comorbidities like rheumatoid arthritis and autoimmune diseases. Hypertension and diabetes were defined as described elsewhere^4^. Estimated glomerular filtration rate (eGFR) was created based on^8^, using blood creatinine levels from DataField 30700. Age and sex were taken from DataFields 21022 and 31, respectively. Urine albumin-creatinine ratio (UACR) was calculated using the formula (Albumin (mg/L)/ Creatinine (µmol/L)) × 8840.17. Albumin and Creatinine Urine levels were taken from DataFields 30500 and 30510, respectively. Statins were defined based on medications codes 1140861958, 1141188146, 1140881748, 1140888648, 1140861970, 1140888594, 1140864592, 1141146234, 1141146138, 1141192410 and 1141192414 in DataField 20003.

## Instrumental variables for IL-6R signaling

We used SNPs in the *IL6R*  region (±300kB around the gene and clumped at pairwise r2<0.1) as instrumental variables that have been validated to mimic the effect of IL-6R signaling and its inhibition (change in CRP, circulating IL-6 and soluble IL-6R levels) and have been described before in detail (**Supplemental Table 2**)^9,10^. The F-statistic ranged from 4.4-458.2 indicating mostly strong genetic instruments (F-statistic >10, **Supplemental Table 2**). These SNPs have been selected based on genome-wide significant association with CRP levels (a known downstream biomarker of IL-6R signaling) by meta-analysis of a genome-wide association study (GWAS) in 204,402 participants of European ancestry in the Cohorts for Heart and Aging Research in Genomic Epidemiology (CHARGE) Consortium^11^ and a GWAS in 318,279 White British individuals in the UKB^12^. To avoid weak instrument bias^13^, association estimates were taken from the CHARGE GWAS. As indicated in the respective statistical analysis and results section, SNPs were either taken as separate instrumental variables or combined into a score and calculated for UKB using pgs-calc (available at:  <https://github.com/lukfor/pgs-calc>)^14^. In 428,407 White UKB individuals the variance explained was 0.15% between the *IL6R* score and CRP concentrations (squared Pearson correlation coefficient between the genetic score and the exposure).

## Instrumental variables for Lp(a) concentrations

We used a previously described weighted score composed of 43 SNPs in the *LPA* region (660kb window) that were conditionally and genome-wide significantly associated with Lp(a) in the CHD Exome+ Consortium participants free from coronary heart disease (no overlap with UKB and exclusion of variants at r2>0.4)^15,16^. It has been validated to explain 51-63% of variance in Lp(a) concentrations and is causally associated with coronary heart disease^16^. The score was downloaded by PGS catalogue (PGS000667 available at: <https://www.pgscatalog.org/score/PGS000667/> ) and calculated for UKB samples using pgs-calc (available at: <https://github.com/lukfor/pgs-calc>)^14^. In 428,407 White UKB individuals the variance explained was 62.39% between the *LPA* score and Lp(a) concentrations (squared Pearson correlation coefficient between the genetic score and the exposure).

To check in reverse two-sample MR if Lp(a) concentrations causally influence IL-6 concentrations we used 14 SNPs in the *LPA* region as separate instrumental variables (**Supplemental Table 4**). The F-statistic ranged from 9.8-1769.8 indicating mostly strong genetic instruments (F-statistic >10, **Supplemental Table 4**). These SNPs were derived from a meta-GWAS on inverse normally transformed Lp(a) concentrations (n=13,781, no overlap with UKB) by Mack et al.^17^. To select the instrumental variables, the genome-wide significantly and conditionally independently associated SNPs  (taken from s6 of^17^, with β and standard error (SE) taken from the columns for single SNP analysis, fixed effects model, on inverse normally transformed scale) were filtered for their location ±250kb around the *LPA* gene and they were clumped at r2<0.1 using the TwoSampleMR package^18^ using European ancestry as reference.

## Colocalization analysis

Colocalization analysis was performed as a sensitivity analysis for MR^19^ in the *IL6R* locus to compare the genetic association signal of CRP as a proxy for IL-6R signaling and Lp(a) using the coloc package for R^20^. The following hypotheses were tested with an Approximate Bayes Factor colocalization giving out posterior probabilities for the hypotheses: H0: neither CRP concentrations (trait 1) nor Lp(a) concentrations (trait 2) have a genetic association in the region, H1: only trait 1 has a genetic association in the region, H2: only trait 2 has a genetic association in the region, H3: both traits are associated, but with different causal variants and H4: both traits are associated and share a single causal variant. Colocalization was declared if the posterior probability for a shared causal variant (H4) was >80%. We used summary statistics (restricted to ±300kB around the *IL6R* gene) of the GWAS on CRP in the CHARGE consortium^11^ (derived from openGWAS (ieu-b-35) database^21^) and of the in-house GWAS on Lp(a) in UKB.

# Supplemental Figures

Supplemental Figure 1. IL-6R signaling is causally associated with Lp(a). Scatterplot of the association of instrumental variables with IL-6R signaling (proxied by natural log transformed CRP concentrations) and Lp(a) concentrations (RINTed = rank inverse normally transformed). The error bars around the instrumental variables indicate 95% confidence intervals. The line indicates the inverse-variance weighted causal estimate. (**A)** Two-sample MR with 24 SNPs in the *IL6R* region (± 300kB) and **(B)** sensitivity analysis with 7 SNPs within the *IL6R* gene.

Supplemental Figure 2. Leave-one-out estimates of the two-sample MR on Lp(a). The exposure is IL-6R signaling (proxied by CRP) using 24 instrumental variables. The estimate labelled with its respective rsID includes all variants except the labeled variant. The inverse-variance weighted estimate including all variants (labeled “IVW estimate”) is also provided for reference.

Supplemental Figure 3. Lp(a) is not causally associated with IL-6 plasma levels but with CHD. Scatterplot of the association of instrumental variables for Lp(a) concentrations (RINTed = rank inverse normally transformed) within the *LPA* locus in **(A)** with IL-6 concentrations (NPX = normalized protein expression) and in **(B)** with coronary heart disease (CHD). The error bars around the instrumental variables indicate 95% confidence intervals. The line indicates the inverse-variance weighted causal estimate.

# Supplemental Tables

| **Supplemental Table 1.** Definition of cardiovascular disease and comorbidities in UK Biobank (UKB) | | | | |
| --- | --- | --- | --- | --- |
| Phenotype | **Source** | **Data Field** | **Corresponding code** |  |
| Cardiovascular Disease* | Date of first reported occurrences | 131298, 131300, 131302, 131304, 131306, 131366, 131368, 131382 | ICD10 code: I21-25; I63, I64, I71 |  |
|  | Date of first operative procedure in hospital inpatients record | 41282 | OPCS4 code: K40, K41, K42, K43, K44, K45, K46, K49, K50, L18, L19, L27, L28 in DataField 41272 |  |
|  | Year of self-reported operation | 20010 | UKB self-reported code: 1070, 1095, 1523, 1104 in DataField 20004 |  |
| Autoimmune Diseases  (prevalent cases) | Diagnosis in hospital inpatients | 41270 | ICD10 code: M05, M06, M07, I00, G35, G70, K50, K51, M30, M32, M33, M45, M88, L95 |  |
| Rheumatoid arthritis (prevalent cases) | Diagnosis in hospital inpatients | 41270 | ICD10 code: M05, M06 |  |

ICD10, International Classification of Disease version 10; OPCS4, Office of Population Censuses and Surveys Classification of Interventions and Procedures, version4

*Cardiovascular events are mapped based on the first reported diagnosis or operative procedure. Incident cases are defined using age as timescale, as described in the Methods. Individuals who experienced an event before enrollment are counted as prevalent cases for baseline characteristics tables.

| Supplemental Table 2. Instrumental variables for IL-6R signaling (proxied by natural log transformed CRP concentrations) in the *IL6R* locus. | | | | | | | |
| --- | --- | --- | --- | --- | --- | --- | --- |
| **SNP** | **Effect allele** | **Other allele** | **β** | **se** | **p-value** | **p-value META** | **Fstat** |
| rs78035035 | a | c | 0.052 | 0.0248 | 0.036 | 1.43e-08 | 4.4 |
| rs112203594 | a | c | 0.0591 | 0.024 | 0.014 | 2.09e-08 | 6.1 |
| rs3738028 | a | c | 0.0121 | 0.0047 | 0.010 | 1.19e-09 | 6.6 |
| rs116141616* | a | g | 0.0614 | 0.0197 | 0.002 | 1.76e-08 | 9.7 |
| rs12406117 | a | g | 0.0136 | 0.0043 | 0.002 | 3.83e-09 | 10.0 |
| rs144029367 | c | t | 0.0752 | 0.0185 | 4.81e-05 | 5.43e-10 | 16.5 |
| rs61806853 | t | c | 0.0456 | 0.0112 | 4.67e-05 | 1.23e-18 | 16.6 |
| rs76289529 | c | t | 0.0715 | 0.0171 | 2.9e-05 | 4.78e-18 | 17.5 |
| rs6698385 | g | a | 0.0216 | 0.0051 | 2.28e-05 | 3.74e-41 | 17.9 |
| rs145262901* | g | a | 0.0844 | 0.0183 | 3.99e-06 | 2.38e-09 | 21.3 |
| rs145909430* | t | c | 0.1396 | 0.0272 | 2.86e-07 | 2.86e-34 | 26.3 |
| rs41269913 | c | t | 0.0779 | 0.0147 | 1.16e-07 | 2.28e-13 | 28.1 |
| rs77994623 | t | c | 0.0332 | 0.0061 | 5.25e-08 | 1.5e-58 | 29.6 |
| rs183641528 | g | a | 0.1034 | 0.0178 | 6.29e-09 | 1.62e-26 | 33.7 |
| rs113580743* | a | g | 0.0708 | 0.0112 | 2.59e-10 | 1.09e-23 | 40.0 |
| rs56100876 | g | a | 0.18 | 0.0262 | 6.41e-12 | 3.27e-42 | 47.2 |
| rs12735458 | a | g | 0.126 | 0.0183 | 5.77e-12 | 4.53e-20 | 47.4 |
| rs73026617 | t | c | 0.0474 | 0.0068 | 3.16e-12 | 1.69e-42 | 48.6 |
| rs7525477* | a | g | 0.0382 | 0.0051 | 6.88e-14 | 1.35e-38 | 56.1 |
| rs11264224 | a | c | 0.0465 | 0.0057 | 3.41e-16 | 1.6e-49 | 66.6 |
| rs16836054 | a | g | 0.0453 | 0.0054 | 4.91e-17 | 1.51e-75 | 70.4 |
| rs12059682 | c | t | 0.0441 | 0.0049 | 2.26e-19 | 2.11e-77 | 81.0 |
| rs12083537* | a | g | 0.0643 | 0.0053 | 7.14e-34 | 3.03e-156 | 147.2 |
| rs2228145* | a | c | 0.0899 | 0.0042 | 1.21e-101 | 3e-307 | 458.2 |

* SNP used for sensitivity analysis only including SNPs within the *IL6R* gene.

β, se (standard error) and p-value from the CHARGE consortium as listed in Table S1 in^10^ excluding rs3766925 and rs34693607 since they are palindromic SNPs.

p-value META: p-value from the meta-analysis of CHARGE and UKB data as explained in^9,10^.

Fstat: F-statistic calculated based on formula 8.3 page 124 in^22^

| **Supplemental Table 3**. Population-specific allele frequencies from the 1000 Genomes Project extracted from the NCBI dbSNP database. | | | |
| --- | --- | --- | --- |
| **Population in**  **1000 Genome** | **Sample Size** | **Reference Allele** | **Alternate Allele** |
|  |  |  |  |
| Global | 5,008 | A=0.7069 | C=0.2931 |
| [African](https://www.ncbi.nlm.nih.gov/biosample/SAMN07486022) | 1,322 | A=0.9130 | C=0.0870 |
| [East](https://www.ncbi.nlm.nih.gov/biosample/SAMN07486024) Asian | 1,008 | A=0.6766 | C=0.3234 |
| Europe | 1,006 | A=0.6402 | C=0.3598 |
| [South](https://www.ncbi.nlm.nih.gov/biosample/SAMN07486027) Asian | 978 | A=0.700 | C=0.300 |
| [American](https://www.ncbi.nlm.nih.gov/biosample/SAMN07488242) | 694 | A=0.464 | C=0.536 |

Source dbSNP [Internet]. Bethesda (MD): National Library of Medicine (US), National Center for Biotechnology Information; 1998, available at: <https://www.ncbi.nlm.nih.gov/snp/rs2228145#frequency_tab>, last accessed on 2026-02-13

| Supplemental Table 4. Instrumental variables for Lp(a) concentrations in the *LPA* locus. | | | | | | |
| --- | --- | --- | --- | --- | --- | --- |
| **SNP** | **Effect allele** | **Other allele** | **β** | **se** | **p-value** | **Fstat** |
| rs118039278 | A | G | 1.22 | 0.029 | <1e-200 | 1769.8 |
| rs186696265 | T | C | 2.02 | 0.062 | <1e-200 | 1061.5 |
| rs6938647 | C | A | 0.37 | 0.016 | 7.48e-119 | 534.8 |
| rs41272114 | C | T | 0.78 | 0.04 | 4.74e-86 | 380.2 |
| rs12529361 | C | T | 0.30 | 0.017 | 5.57e-69 | 311.4 |
| rs41269133 | T | C | 0.33 | 0.021 | 5.42e-55 | 246.9 |
| rs142126734 | A | G | 0.44 | 0.03 | 8.52e-48 | 215.1 |
| rs62441903 | A | G | 0.82 | 0.069 | 4.86e-33 | 141.2 |
| rs41259144 | C | T | 0.85 | 0.084 | 4.45e-24 | 102.4 |
| rs73596816 | A | G | 0.66 | 0.075 | 1.53e-18 | 77.4 |
| rs116850263 | C | T | 0.57 | 0.069 | 1.01e-16 | 68.2 |
| rs76000021 | T | C | 0.41 | 0.057 | 4.7e-13 | 51.7 |
| rs79246098 | C | T | 0.28 | 0.07 | 7.53e-05 | 16.0 |
| rs2457557 | C | T | 0.05 | 0.016 | 0.001 | 9.8 |

β, se (standard error) and p-value from the GWAS meta-analysis on rank inverse normally transformed Lp(a) concentrations fixed effects model as listed in Table s6 of^17^.

Fstat: F-statistic calculated based on formula 8.3 on page 124 in^22^.

| Supplemental Table 5. Results of the two-sample MR on Lp(a) concentrations using 24 SNPs in the IL6R region as instrumental variables. | | | |
| --- | --- | --- | --- |
| **Method** | **β** | **95% confidence interval** | **p-value** |
| IVW | 0.064 | 0.042,0.085 | <0.001 |
| Simple median | 0.058 | 0.027,0.090 | <0.001 |
| Weighted median | 0.077 | 0.051,0.104 | <0.001 |
| MR-Egger | 0.072 | 0.032,0.113 | 0.001 |
| MR-Egger intercept | -0.001 | -0.003,0.002 | 0.616 |
| MR-PRESSO | 0.064 |  | <0.001 |
| MR-PRESSO outlier-corrected | NA | NA | NA |

IVW, inverse-variance weighted; MR, Mendelian Randomization

| Supplemental Table 6. Results of the two-sample MR on IL-6 plasma concentrations using 14 SNPs in the *LPA* region as instrumental variables. | | | |
| --- | --- | --- | --- |
| **Method** | **β** | **95% confidence interval** | **p-value** |
| IVW | 0.015 | -0.001,0.032 | 0.066 |
| Simple median | 0.005 | -0.001,0.032 | 0.70 |
| Weighted median | 0.005 | -0.020,0.030 | 0.70 |
| MR-Egger | 0.013 | -0.013,0.038 | 0.34 |
| MR-Egger intercept | 0.002 | -0.013,0.017 | 0.79 |

IVW, inverse-variance weighted; MR, Mendelian Randomization

| Supplemental Table 7. Baseline characteristics of the UK Biobank cohort and the subset of participants included in the observational analysis. | | |
| --- | --- | --- |
|  | **UKB Dataset*** | **UKB Subset**† |
| Sample Size, n | 471,607 | 44,233 |
| **Demographics** |  |  |
| Sex, men n (%) | 214,726 (45.5) | 20,401 (46.1) |
| Age, years | 58.00 (50.00- 63.00) | 59.00 (51.00- 64.00) |
| Body mass index, kg/m^2^ | 26.72 (24.13- 29.87) | 26.75 (24.16- 29.87) |
| Smoking Status, n (%) |  |  |
| Never | 253,509 (53.9) | 23,579 (53.5) |
| Previous | 167,069 (35.6) | 15,897 (36.1) |
| Current | 49,362 (10.5) | 4,618 (10.5) |
| **Baseline clinical conditions** |  |  |
| Hypertension, n (%) | 259,114 (55.0) | 24,686 (55.8) |
| Diabetes, n (%) | 21,175 (4.8) | 2,132 (5.0) |
| Cardiovascular disease, n (%) | 23,330 (4.9) | 2,459 (5.6) |
| Autoimmune disease, (%) | 14,358 (3.0) | 1,688 (3.8) |
| Rheumatoid arthritis, n (%) | 4,446 (0.9) | 464 (1.0) |
| **Lipid profile** |  |  |
| Total Cholesterol, mmol/L | 5.67 (4.93- 6.44) | 5.63 (4.87- 6.42) |
| LDL-cholesterol, mg/dL | 136.37 (114.22- 159.64) | 135.14 (112.67- 159.22) |
| HDL-cholesterol, mmol/L | 1.40 (1.18- 1.68) | 1.40 (1.17- 1.68) |
| Triglyceride, mmol/L | 1.49 (1.05- 2.15) | 1.49 (1.05- 2.15) |
| Lp(a), nmol/L | 18.74 (7.40- 72.72) | 18.77 (7.39- 72.00) |
| **Laboratory parameters** |  |  |
| eGFR, mL/min/1.73m^2^ | 92.96 (83.17- 100.04) | 92.61 (82.44- 99.72) |
| UACR, mg/g‡ | 9.29 (5.80- 18.19) | 9.43 (5.88- 19.02) |
| CRP, mg/L | 1.33 (0.66- 2.75) | 1.34 (0.66- 2.78) |
| Interleukin-6 | 0.00 (-0.46- 0.55) | 0.00 (-0.46- 0.54) |
| *IL6R* mutations (rs2228145), n (%) |  |  |
| Wild type (A/A) | 160,353 (35.0) | 15,571 (35.2) |
| Heterozygous (A/C) | 220,413 (48.1) | 21,257 (48.1) |
| Homozygous (C/C) | 77,415 (16.9) | 7,405 (16.7) |
| **Medication** |  |  |
| Statins, n (%) | 77,137 (16.4) | 7,827 (17.7) |

Values for continuous data are presented as median (25^th^-75^th^ percentiles) and for categorical measures as count (percentage). LDL, low-density lipoprotein; HDL, high-density lipoprotein; Lp(a), lipoprotein(a); eGFR, estimated glomerular filtration rate; UACR, urine albumin-creatinine ratio; CRP, high-sensitivity C-reactive protein; IL6R, interleukin-6 receptor. Cardiovascular disease are prevalent events (**Supplemental Table 1**) before enrollment.

* Overall UK Biobank participants of White ancestry (DataField 21000, entries “British”, “Irish” or “Any other White background”, not withdrawn as of August 2025).

† Subset of individuals with available data on circulating IL-6, Lp(a) and *IL6R* mutation (rs2228145).

‡UACR was calculated as: (Albumin (mg/L)/ Creatinine (µmol/L) ) × 8840.17.

| **Supplemental Table 8.** Baseline characteristics of participants in the UK Biobank cohort (n=471,607) stratified based on SNP in *IL6R* (rs2228145). | | | | |
| --- | --- | --- | --- | --- |
|  | **Wild type (A/A)** | **Heterozygous (A/C)** | **Homozygous (C/C)** | **p-value** |
| Sample Size, n (%)* | 160,353 (34) | 220,413 (47) | 77,415 (16) |  |
| **Demographics** |  |  |  |  |
| Sex, men n (%) | 73,283 (45.7) | 100,660 (45.7) | 35,483 (45.8) | 0.73 |
| Age, years | 58.00 (50.00- 63.00) | 58.00 (50.00- 63.00) | 58.00 (51.00- 63.00) | **0.014** |
| Body mass index, kg/m^2^ | 26.71 (24.13- 29.84) | 26.71 (24.12- 29.87) | 26.72 (24.13- 29.86) | 0.91 |
| Smoking Status, n (%) |  |  |  | 0.070 |
| Never | 85,858 (53.5) | 118,648 (53.8) | 41,823 (54.0) |  |
| Previous | 57,034 (35.6) | 78,043 (35.4) | 27,403 (35.4) |  |
| Current | 16,883 (10.5) | 22,968 (10.4) | 7,899 (10.2) |  |
| **Baseline clinical conditions** |  |  |  |  |
| Hypertension, n (%) | 88,048 (54.9) | 121,103 (55.0) | 42,731 (55.2) | 0.38 |
| Diabetes, n (%) | 7,254 (4.7) | 9,963 (4.7) | 3,412 (4.6) | 0.38 |
| Cardiovascular disease, n (%) | 8,032 (5.0) | 10,866 (4.9) | 3,750 (4.8) | 0.14 |
| Autoimmune disease, n (%) | 5,053 (3.2) | 6,592 (3.0) | 2,181 (2.8) | **<0.001** |
| Rheumatoid arthritis, n (%) | 1,527 (1.0) | 2,075 (0.9) | 662 (0.9) | 0.053 |
| **Medication** |  |  |  |  |
| Statins, n (%) | 26,521 (16.5) | 35,907 (16.3) | 12,568 (16.2) | 0.067 |

Values for continuous data are presented as median (25^th^-75^th^ percentiles) and for categorical measures as count (percentage). Cardiovascular disease cases are prevalent CVD events (as defined in **Supplemental Table 1**) that occurred before enrollment. Statistically significant p-values are depicted in bold.

*Sample size within each stratified group is expressed as count (percentage), where percentage represents the proportion relative to the total UK Biobank cohort (n=471,607).

| **Supplemental Table 9.** Trends in lipid profiles and laboratory parameters in the UK Biobank cohort (n=471,607) stratified based on SNP in *IL6R* (rs2228145). | | | | |
| --- | --- | --- | --- | --- |
|  | **Wild type (A/A)** | **Heterozygous (A/C)** | **Homozygous (C/C)** | ***P _trend_*** |
| Sample Size, n (%)* | 160,353 (34) | 220,413 (47) | 77,415 (16) |  |
| **Lipid profile** |  |  |  |  |
| Total Cholesterol, mmol/L | 5.66 (4.92- 6.43) | 5.67 (4.93- 6.44) | 5.68 (4.94- 6.46) | **<0.001** |
| LDL-cholesterol, mg/dL | 136.22 (114.07- 159.34) | 136.37 (114.30- 159.76) | 136.57 (114.42- 159.72) | **0.012** |
| HDL-cholesterol, mmol/L | 1.40 (1.17- 1.68) | 1.40 (1.18- 1.68) | 1.41 (1.17- 1.69) | **0.002** |
| Triglyceride, mmol/L | 1.49 (1.05- 2.15) | 1.49 (1.05- 2.15) | 1.49 (1.05- 2.16) | 0.27 |
| Lp(a), nmol/L | 18.90 (7.41- 73.81) | 18.79 (7.40- 72.30) | 18.40 (7.30- 71.89) | **0.004** |
| **Laboratory parameters** |  |  |  |  |
| eGFR, mL/min/1.73m^2^ | 92.93 (83.12- 99.99) | 93.00 (83.20- 100.07) | 92.92 (83.21- 99.99) | 0.54 |
| UACR, mg/g | 9.30 (5.80- 17.95) | 9.27 (5.79- 18.26) | 9.28 (5.79- 18.30) | 0.48 |
| CRP, mg/L | 1.44 (0.71- 2.95) | 1.31 (0.65- 2.73) | 1.16 (0.58- 2.41) | **<0.001** |
| Interleukin-6 | -0.14 (-0.59- 0.39) | 0.01 (-0.43- 0.55) | 0.22 (-0.22- 0.79) | **<0.001** |

Values for continuous data are presented as median (25^th^-75^th^ percentiles) and for categorical measures as count (percentage). LDL, low-density lipoprotein; HDL, high-density lipoprotein; Lp(a), lipoprotein(a); eGFR, estimated glomerular filtration rate; UACR, urine albumin-creatinine ratio; CRP, high-sensitivity C-reactive protein. Statistically significant p-values are depicted in bold.

*Sample size within each stratified group is expressed as count (percentage), where percentage represents the proportion relative to the total UK Biobank cohort (n=471,607).

| Supplemental Table 10. Baseline table stratified into four groups based on median IL-6R and LPA score groups. | | | | |
| --- | --- | --- | --- | --- |
|  | **Both scores > median** | **IL-6R score ≤ median** | **LPA score ≤ median** | **Both scores ≤ median** |
| Sample Size, n | 113,539 | 113,542 | 115,551 | 115,549 |
| **Demographics** |  |  |  |  |
| Sex, men n (%) | 51,809 (45.6) | 51,933 (45.7) | 52,892 (45.8) | 52,792 (45.7) |
| Age, years | 58.00 (50.00- 63.00) | 58.00 (50.00- 63.00) | 58.00 (50.00- 63.00) | 58.00 (51.00- 63.00) |
| Body mass index, kg/m^2^ | 26.71 (24.12- 29.85) | 26.71 (24.11- 29.86) | 26.74 (24.15- 29.88) | 26.71 (24.12- 29.84) |
| Smoking Status, n (%) |  |  |  |  |
| Never | 60,690 (53.6) | 61,306 (54.2) | 61,982 (53.8) | 62,351 (54.2) |
| Previous | 40,456 (35.8) | 40,176 (35.5) | 41,052 (35.6) | 40,796 (35.4) |
| Current | 11,985 (10.6) | 11,653 (10.3) | 12,129 (10.5) | 11,983 (10.4) |
| **Baseline clinical conditions** |  |  |  |  |
| Hypertension, n (%) | 62661 (55.2) | 62,770 (55.3) | 63,091 (54.6) | 63,360 (54.9) |
| Diabetes, n (%) | 5,196 (4.8) | 5,079 (4.7) | 5,197 (4.7) | 5,157 (4.7) |
| Cardiovascular disease, n (%) | 6,095(5.4) | 5,924 (5.2) | 5,323 (4.6) | 5,306 (4.6) |
| Autoimmune disease, n (%) | 3,560 (3.1) | 3,376 (3.0) | 3,528 (3.1) | 3,362 (2.9) |
| Rheumatoid arthritis, n (%) | 1,119 (1.0) | 1,040 (0.9) | 1,095 (0.9) | 1,010 (0.9) |
| Lipid profile |  |  |  |  |
| Total Cholesterol, mmol/L | 5.68 (4.93- 6.46) | 5.70 (4.95- 6.47) | 5.65 (4.90- 6.41) | 5.65 (4.93- 6.42) |
| LDL-cholesterol, mg/dL | 136.88 (114.65- 160.30) | 137.22 (114.88- 160.77) | 135.64 (113.53- 158.60) | 135.72 (113.92- 158.79) |
| HDL-cholesterol, mmol/L | 1.40 (1.18- 1.68) | 1.41 (1.18- 1.68) | 1.40 (1.17- 1.68) | 1.41 (1.18- 1.68) |
| Triglyceride, mmol/L | 1.47 (1.04- 2.13) | 1.47 (1.04- 2.13) | 1.51 (1.06- 2.18) | 1.51 (1.07- 2.17) |
| Lp(a), nmol/L | 64.40 (22.40- 146.90) | 63.50 (21.84- 145.60) | 8.70 (4.50- 16.90) | 8.70 (4.44- 16.80) |
| **Laboratory parameters** |  |  |  |  |
| eGFR | 92.88 (82.94- 99.95) | 92.99 (83.22- 99.99) | 93.00 (83.24- 100.10) | 92.98 (83.26- 100.06) |
| UACR, mg/g | 9.29 (5.81- 18.11) | 9.24 (5.78- 18.11) | 9.32 (5.79- 17.99) | 9.28 (5.79- 18.41) |
| CRP, mg/L | 1.40 (0.69- 2.89) | 1.25 (0.62- 2.60) | 1.42 (0.70- 2.92) | 1.25 (0.62- 2.60) |
| Interleukin-6 | -0.08 (-0.53- 0.45) | 0.08 (-0.37- 0.64) | -0.10 (-0.55- 0.44) | 0.07 (-0.37- 0.63) |
| **Medication** |  |  |  |  |
| Statins, n (%) | 19,931 (17.6) | 19,398 (17.1) | 17,719 (15.3) | 17,948 (15.5) |

Values for continuous data are presented as median (25^th^-75^th^ percentiles) and for categorical measures as count (percentage). LDL, low-density lipoprotein; HDL, high-density lipoprotein; Lp(a), lipoprotein(a); eGFR, estimated glomerular filtration rate; UACR, urine albumin-creatinine ratio; CRP, high-sensitivity C-reactive protein. Cardiovascular disease are prevalent events (**Supplemental Table 1**) before enrollment.

# References

1.	Bycroft C, Freeman C, Petkova D, et al. The UK Biobank resource with deep phenotyping and genomic data. *Nature* 2018; **562**(7726): 203-9.

2.	McCarthy S, Das S, Kretzschmar W, et al. A reference panel of 64,976 haplotypes for genotype imputation. *Nat Genet* 2016; **48**(10): 1279-83.

3.	Huang J, Howie B, McCarthy S, et al. Improved imputation of low-frequency and rare variants using the UK10K haplotype reference panel. *Nat Commun* 2015; **6**: 8111.

4.	Di Maio S, Lamina C, Coassin S, et al. Lipoprotein(a) and SARS-CoV-2 infections: Susceptibility to infections, ischemic heart disease and thromboembolic events. *J Intern Med* 2022; **291**(1): 101-7.

5.	Sun BB, Chiou J, Traylor M, et al. Plasma proteomic associations with genetics and health in the UK Biobank. *Nature* 2023; **622**(7982): 329-38.

6.	Schachtl-Riess JF, Kheirkhah A, Grüneis Ra. Frequent LPA KIV-2 Variants Lower Lipoprotein(a) Concentrations and Protect Against Coronary Artery Disease. *J Am Coll Cardiol* 2021; **78**(5): 437-49.

7.	van der Harst P, Verweij N. Identification of 64 Novel Genetic Loci Provides an Expanded View on the Genetic Architecture of Coronary Artery Disease. *Circ Res* 2018; **122**(3): 433-43.

8.	Levey AS, Stevens LA, Schmid CH, et al. A New Equation to Estimate Glomerular Filtration Rate. *Ann Intern Med* 2009; **150**(9): 604.

9.	Georgakis MK, Malik R, Burgess S, Dichgans M. Additive Effects of Genetic Interleukin‐6 Signaling Downregulation and Low‐Density Lipoprotein Cholesterol Lowering on Cardiovascular Disease: A 2×2 Factorial Mendelian Randomization Analysis. *Journal of the American Heart Association* 2022; **11**(1): e023277.

10.	Georgakis MK, Malik R, Richardson TG, et al. Associations of genetically predicted IL-6 signaling with cardiovascular disease risk across population subgroups. *BMC Med* 2022; **20**(1): 245.

11.	Ligthart S, Vaez A, Võsa U, et al. Genome Analyses of >200,000 Individuals Identify 58 Loci for Chronic Inflammation and Highlight Pathways that Link Inflammation and Complex Disorders. *Am J Hum Genet* 2018; **103**(5): 691-706.

12.	Sinnott-Armstrong N, Tanigawa Y, Amar D, et al. Genetics of 35 blood and urine biomarkers in the UK Biobank. *Nat Genet* 2021; **53**(2): 185-94.

13.	Burgess S, Davies NM, Thompson SG. Bias due to participant overlap in two-sample Mendelian randomization. *Genet Epidemiol* 2016; **40**(7): 597-608.

14.	Forer L, Taliun D, LeFaive J, et al. Imputation Server PGS: an automated approach to calculate polygenic risk scores on imputation servers. *Nucleic Acids Res* 2024; **52**(W1): W70-w7.

15.	Trinder M, Uddin MM, Finneran P, Aragam KG, Natarajan P. Clinical Utility of Lipoprotein(a) and LPA Genetic Risk Score in Risk Prediction of Incident Atherosclerotic Cardiovascular Disease. *JAMA Cardiol* 2020; **6**(3): 1-9.

16.	Burgess S, Ference BA, Staley JR, et al. Association of LPA Variants With Risk of Coronary Disease and the Implications for Lipoprotein(a)-Lowering Therapies: A Mendelian Randomization Analysis. *JAMA Cardiol* 2018; **3**(7): 619-27.

17.	Mack S, Coassin S, Rueedi R, et al. A genome-wide association meta-analysis on lipoprotein (a) concentrations adjusted for apolipoprotein (a) isoforms. *J Lipid Res* 2017; **58**(9): 1834-44.

18.	Hemani G, Tilling K, Davey Smith G. Orienting the causal relationship between imprecisely measured traits using GWAS summary data. *PLoS Genet* 2017; **13**(11): e1007081.

19.	Zuber V, Grinberg NF, Gill D, et al. Combining evidence from Mendelian randomization and colocalization: Review and comparison of approaches. *Am J Hum Genet* 2022; **109**(5): 767-82.

20.	Giambartolomei C, Vukcevic D, Schadt EE, et al. Bayesian test for colocalisation between pairs of genetic association studies using summary statistics. *PLoS Genet* 2014; **10**(5): e1004383.

21.	Lyon MS, Andrews SJ, Elsworth B, Gaunt TR, Hemani G, Marcora E. The variant call format provides efficient and robust storage of GWAS summary statistics. *Genome Biol* 2021; **22**(1): 32.

22.	Burgess S, Thompson, SG. Mendelian Randomization: Methods for Causal Inference Using Genetic Variants. 2nd Edition ed. New York: Chapman and Hall/CRC; 2021.
